# Myocarditis: An Interleukin-1-Mediated Disease?

**DOI:** 10.3389/fimmu.2018.01335

**Published:** 2018-06-13

**Authors:** Giacomo De Luca, Giulio Cavalli, Corrado Campochiaro, Moreno Tresoldi, Lorenzo Dagna

**Affiliations:** ^1^Unit of Immunology, Rheumatology, Allergy and Rare Diseases (UnIRAR), IRCCS San Raffaele Scientific Institute, Vita-Salute San Raffaele University, Milan, Italy; ^2^Department of Medicine, Radboud University Medical Center, Nijmegen, Netherlands; ^3^Unit of General Medicine and Advanced Care, IRCCS San Raffaele Hospital and Scientific Institute, Milan, Italy

**Keywords:** autoimmune myocarditis, interleukin-1, auto-inflammatory diseases, heart failure, anti-cytokine therapy

## Introduction

Myocarditis is defined by WHO as an inflammatory disease affecting the myocardium, diagnosed by endomyocardial biopsy (EMB) using established histological, immunological, and immunohistochemical criteria; it may be idiopathic, infectious, or autoimmune (1–4). A wide variety of histological myocarditis patterns have been described according to the diverse etiologies and to the stage of the disease at the time of EMB ascertainment. Viral infections represent the most common cause in Europe and North America (5). Different viral genomes can be detected in the myocardium of patients with myocarditis and dilated cardiomyopathy (DCM) using molecular techniques (6–14). When no infectious agents are identified on EMB and other known causes are excluded, autoimmune myocarditis (AMy) is the presumed etiology (15). These so-called “autoimmune myocarditis” or “virus-negative myocarditis” may occur as a distinct disease with exclusive cardiac involvement, or in the context of systemic autoimmune or inflammatory disorders with extracardiac involvement (5, 16–23).

It is likely that several etiologic types of myocarditis confluence in a common immune-mediated pathogenic process leading to chronic inflammation and tissue damage. Irrespective of triggering agents, acute inflammation may progress to subacute and chronic stages and eventually result in tissue remodeling, fibrosis, contractile dysfunction, and finally DCM (4, 6, 8, 24–29). Besides contractile dysfunction, both early myocardial inflammation and late fibrotic changes play a critical role in the development of the arrhythmic burden, which makes myocarditis one of the leading causes of sudden death (19, 27–31). Clearly, treatment interventions effectively curbing the acute inflammatory process at an early stage can prevent late cardiac remodeling and improve patient’s outcome. Emerging evidence on the pathogenic mechanisms underlying cardiac inflammation is paving the way to novel, promising treatment strategies for myocarditis.

## Pathogenesis

Both autoimmune and innate inflammatory responses are involved in the pathogenesis of myocarditis and its sequelae; however, the specific contribution of either mechanism is not completely understood. As most of immune-mediated diseases, VNM is considered a multifactorial entity, with several immunologic mechanisms contributing to disease development and progression. Environmental triggers of myocardium damage lead to local inflammation and exposure of cryptic self-antigens: excessive innate and myocardium-specific immune responses ensue in genetically predisposed individuals (16, 31–35) and result in a self-sustained autoimmune or inflammatory cycle independent of the original triggering agent.

Traditionally, persistent autoimmune responses have been postulated to underlie the progression from myocarditis to DCM. Histologically, a lymphocytic infiltrate characterizes VNM, which is historically considered a CD4+ T cells mediated disease. Accordingly, transfer of CD4+ T-cells to severe combined immunodeficient mice induced the disease, while CD4+ T-cells depletion ameliorated experimental AMy (EAM). Additional immune cell-types characterizing cardiac inflammation in EAM include T-helper (Th)-1 and Th-17 cells, neutrophils, eosinophils, and monocytes/macrophages, representing a predominantly adaptive cellularity (32, 34). The role of innate immunity and auto-inflammatory mechanisms in the pathogenesis of myocarditis has traditionally received much less attention. Still, activation of the innate inflammatory response is required to prime and kick-off the adaptive immune response, and persistent inflammation is a critical cause of progressive tissue damage.

## The Auto-Inflammatory Hypothesis

As most tissues, the heart exhibits a stereotyped, highly conserved response to injuries, characterized by an intense inflammatory reaction. This response, eventually leading to myocardial inflammation and heart failure (HF), is mediated by the pro-inflammatory cytokine interleukin (IL)-1, as indicated by both clinical and experimental evidence. IL-1 is an apical pro-inflammatory cytokine: two distinct ligands (IL-1α and IL-1β) with high sequence homology exert their biological activity by binding the IL-1 type-I receptor (IL-1RI), which transduces pro-inflammatory signals leading to the synthesis and expression of hundreds of secondary inflammatory mediators (35). IL-1α is produced by most epithelial cells as a fully active pro-inflammatory mediator, which is released upon cell death or stress in the case of tissue injury, thereby acting as an “alarmin.” Conversely, IL-1β is mostly produced by monocyte-macrophages as an inactive precursor and requires cleavage by an intracellular molecular complex termed “inflammasome” in order to be secreted and exert potent pro-inflammatory effects (35–38). The same cells that produce IL-1α or IL-1β also synthesize various regulatory molecules to curb excessive inflammation (35–38), including the IL-1 receptor antagonist (IL-1Ra). Competitive binding of IL-1Ra to IL-1RI prevents IL-1 signaling and inhibits IL-1-mediated inflammation (35–37).

**Figure 1:**
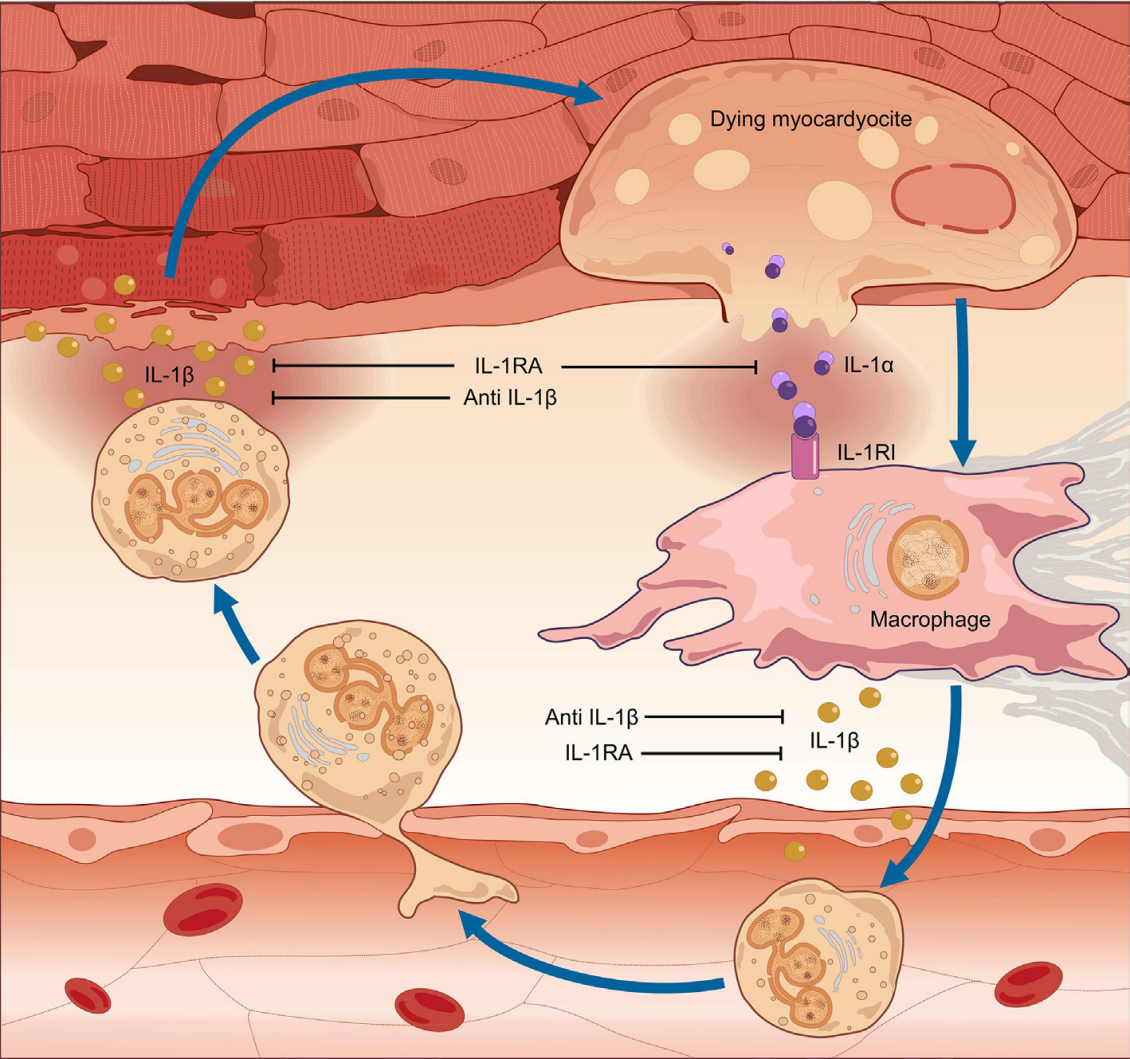
Pathogenesis of myocarditis: the auto-inflammatory hypothesis. In myocarditis, a detrimental inflammatory response leads to cardiac damage, clinically manifested with contractile dysfunction. The pro-inflammatory cytokine interleukin (IL)-1 is of pivotal importance in the pathogenesis of myocardial inflammation. Inflammation of the heart results in myocardial injury. IL-1α is released from the dying myocardiocytes, together with intracellular debris and inflammatory mediators; these in turn activate a molecular complex known as the “inflammasome,” resulting in processing and release of active IL-1β by infiltrating inflammatory cells. Runaway cardiac inflammation ensues, leading to apoptosis of cardiomyocytes and loss of contractile tissue, cardiomyopathy, and heart failure. Given the dual efficacy against myocardial inflammation and contractile dysfunction, prompt pharmacologic inhibition of IL-1 can arrest the progression of uncontrolled inflammation, thus preventing extensive tissue damage and arrhythmic complications and restoring cardiac function.

In myocarditis, IL-1α is released from the dying myocardium together with debris and other inflammatory mediators, and these in turn activate the inflammasomes in nearby cells (35–38). Runaway IL-1-mediated inflammation ensues, progressively causing apoptosis of cardiomyocytes, loss of contractile tissue, fibrosis, cardiomyopathy, HF, and arrhythmic outburst (39) (Figure 1). Intracellular aggregates of apoptosis-associated speck-like protein containing CARD (ASC) and/or caspase-1, indicative of the formation of the inflammasome, can be observed in leukocytes, cardiomyocytes, fibroblasts, and endothelial-cells in EMB samples from patients with acute lymphocytic myocarditis or diagnosed as having myocarditis post-mortem (39). Of note, the number of inflammasome-containing leukocytes per-field correlated with HF severity: patients presenting with HF or with depressed left-ventricular ejection fraction (LVEF) at admission, as well as those who did not experience a significant 6-month LVEF recovery, exhibited a greater number of inflammasomes and inflammasome-containing leukocytes in heart specimens. These data indicate that a stereotyped tissue inflammatory response characterized by the formation of the inflammasome is present and associated with disease severity in acute myocarditis, thus substantiating the critical role of IL-1β in both myocardial inflammation and systolic impairment.

The mechanisms linking IL-1 to impaired contractile function include inhibition of L-type calcium channels, uncoupling of the β-adrenergic receptor (β-AR) from the adenylyl-cyclase (40–46), and transcriptional and posttranslational changes in phospholamban and sarcoplasmic/endoplasmic reticulum calcium ATPase (47). IL-1 also increases nitric oxide (NO)-synthase expression leading to an increased NO activity, which mediates disruption of calcium and β-AR signaling and mitochondrial dysfunction (38, 48–50).

Experimental observations in animal models also confirm the role of IL-1 in inflammatory HF: plasma from advanced HF patients induced a significant systolic and diastolic dysfunction and reduced contractile reserve in mice following a single injection, suggesting the presence of circulating cardiodepressant factors (51, 52). Notably, administration of exogenous IL-1β to mice had comparable effects, while pre-blocking of IL-1 prevented contractile dysfunction induced by HF serum, indicating that cardiodepressant effects are IL-1-mediated (52, 53). This role of IL-1 as a cardiodepressant factor is also described in sepsis (54).

Beyond a direct cardiodepressant activity, several lines of evidence point at a central role of IL-1 signaling in the induction and development of the immune processes associated with acute myocarditis. IL-1 expression is markedly upregulated in experimental models of AMy. Coxsackievirus-induced myocarditis in mice is also characterized by heart infiltration with inflammatory cells secreting IL-1 and TNF-α (55). Persistently elevated IL-1β expression was noted in the chronic stage of myocarditis in a mouse model of post-myocarditis DCM induced by the encephalomyocarditis-virus (56). Consistently, mice deficient for IL-1RI were protected from the development of AM (57), and IL-1Ra administration or expression had beneficial effects in experimental models of inflammatory cardiomyopathy: specifically, delivery of human IL-1Ra *via* plasmid vectors into the hearts of experimental animals with coxsackieviral or AMy decreased myocardial inflammation and reduced mortality (58–61). These findings were paralleled by observations in humans, as increased IL-1β mRNA levels were found in EMBs from patients with viral myocarditis (57) and idiopathic DCM (58). Taken together, these evidences strongly suggest that IL-1 inhibition has the potentiality to simultaneously curb the heart inflammatory response and ameliorate myocardial contractility. Therapeutic agents specifically blocking IL-1 are available and used to treat a broad variety of autoimmune and inflammatory conditions (22, 62–65). Anakinra, the recombinant form of the naturally occurring IL-1Ra, blocks the activity of both IL-1α and IL-1β and is used to treat conditions characterized by IL-1-mediated inflammation. However, despite convincing evidence indicating a pivotal role of IL-1 in the pathogenesis of myocardial inflammation and myocarditis-related systolic dysfunction, the use of IL-1 blocking agents has been only anecdotally reported in this clinical condition.

## Current and Future Therapeutic Perspectives

Symptomatic treatment of HF and of arrhythmias is the mainstay of treatment in myocarditis (66, 67). So far, conventional immunosuppressive drugs (steroids, cyclosporine, and azathioprine), rather than targeted treatments, have been used in order to target the pro-inflammatory mediators of the disease. Single-center randomized trials (RTs) showed some benefits in chronic VNM/DCM (68, 69), in giant-cell myocarditis (70) and in Amy (71). Recently, a single center controlled study demonstrated that combination of azathioprine and steroids can be moderately effective in the treatment of VNM refractory to standard of care (69). In the TIMIC-trial, patients with HF and biopsy-proven VNM were randomized to receive either prednisone 1 mg/kg per day for 4 weeks, followed by 0.33 mg/kg per day for 5-month plus azathioprine 2 mg/kg per day for 6 months, or placebo in addition to conventional therapy for HF. Primary outcome was the 6-month improvement in LVEF. Patients receiving prednisone plus azathioprine exhibited an improvement of LVEF and a decrease in LV volumes compared with baseline, while no patient in the placebo arm showed any improvement in LVEF. These results have been subsequently reproduced by other European observational studies (72, 73). However, a proportion of patients with myocarditis treated with current immunosuppressive strategies showed a complete lack of response, an observation indicating that some critical mechanisms of disease pathogenesis and inflammatory tissue damage are not susceptible to conventional immunosuppression.

## Therapeutic IL-1 Blockade

Given the pivotal role of IL-1 in cardiac inflammation, it is expected that treatment with IL-1 blocking agents would curb inflammation and afford significant clinical benefits in patients with myocarditis. Clinical experience with specific IL-1 inhibition provides unambiguous confirmation to the role of IL-1 in human myocardial inflammation (Table 1). Specifically, in different RTs of ST-elevation myocardial infarction (STEMI) and HF, treatment with anakinra was associated with improved cardiac contractility and function. Also of note, in all these trials, anakinra treatment was associated with marked reductions in serum inflammatory markers. In the recent, massive CANTOS trial of 10,061 patients with previous myocardial infarction and C-reactive protein (CRP) > 2 mg/l, selective blockade of IL-1β with the monoclonal antibody canakinumab conferred protection against recurrent cardiovascular events (74); secondary analyses of results determined that more robust reductions in CRP after the first dose of canakinumab predicted greater clinical benefits, again confirming the role of IL-1 as driving force of cardiac and systemic inflammation (74–80).

**Table 1:** Clinical experience with interleukin-1 inhibition in cardiac diseases.

Study (reference) (year)	Population (*n*)	Study design	Results
VCU-ART (75) (2010)^a^	STEMI (10)	Double-blinded, randomized vs placebo	Anakinra treatment decreased LVESVi and LVEDVi on CMR and TTE (3 months). Trend toward decreased CRP levels correlated with the change in LVESVi and LVEDVi

AIR-HF (51) (2012)	HfrEF and elevated CRP levels (7)	Open-label, single-arm, non-randomized design. Anakinra 100 mg daily for 14 days	Change in aerobic capacity (peak VO2) and ventilatory efficiency (VE/VCO2) between baseline and 14 days

VCUART2 (76) (2013)^a^	STEMI (30)	Double-blind, randomized: vs placebo	No significant change in LVESVi, LVEDVi, and LVEF on cardiac MRI and echocardiography (3 months). Anakinra treatment: blunted the increase in CRP levels

VCUART 3 (2014) https://ClinicalTrials.gov Identifier: *NCT01950299*	STEMI (99)	Double-blind, randomized vs placebo	Results are awaited Primary endpoint: CRP levels in 14 days Secondary: change in LVESVi, LVEF, and new-onset HF (12 months)

CANTOS (74, 80) (2012)^b^	Post-myocardial infarction patients with elevated CRP (10,061)	Double-blind, randomized, multi-center, international, subcutaneous canakinumab 50, 150, or 300 mg every 3 months vs placebo. Median follow-up of 3.8 years	The 150-mg dose met the prespecified multiplicity adjusted threshold for statistical significance for a composite end point of non-fatal myocardial infarction, non-fatal stroke, or cardiovascular death. Nearly all of the risk reduction was observed in non-fatal MI. No significant difference in stroke, cardiovascular mortality, or overall mortality

Ikonomidis et al. (86) (2008)	Rheumatoid arthritis (23)	Double-blind, randomized cross-over trial. Anakinra 100 mg (single injection) vs placebo, baseline compared to 3 h after treatment. After 2 days, the alternate treatment was given^d^	Improvement of FMD, CFR, arterial compliance, resistance, longitudinal strain, circumferential strain, peak twisting, untwisting velocity, LVEF, apoptotic, and oxidative markers in CAD than in non-CAD patients
Ikonomidis et al. (87) (2014)	Rheumatoid arthritis + CAD (60), rheumatoid arthritis + no CAD (20)		

MRC-ILA Heart Study (77) (2014)^c^	NSTEMI (182)	Double-blind, randomized vs placebo	Reduction in CRP levels (area-under-the-curve for CRP) over the first 7 days

D-HART (82) (2014)	HFpEF (12)	Double-blind, randomized, placebo-controlled, crossover trial: anakinra 100 mg daily vs placebo for 14 days and an additional 14 days of the alternate treatment	Improvement in peak oxygen consumption and a significant reduction in plasma CRP levels

D-HART2 (78) (2014)	HFpEF and elevated CRP levels (30)	2:1 double-blind, randomized, placebo-controlled, single center trial: anakinra 100 mg daily or placebo for 12 weeks	Results are awaited

REDHART (2014) https://ClinicalTrials.gov Identifier: NCT01936909	HFrEF with recently decompensated HF (60)	Double-blind, randomized, placebo-controlled: anakinra 100 mg daily for2 or 12 weeks or placebo for 12 weeks, follow-up of 24 weeks	Study completed. Results have yet to be published Primary outcome measure: aerobic exercise capacity (peak VO2) and ventilator efficiency at 2 weeks (follow-up = 24 weeks) Secondary: survival free of hospital admissions

ADHF (79) (2014)	HFrEF, acute decompensation, and elevated CRP levels (30)	Double-blind, randomized, placebo-controlled: anakinra 100 mg twice daily for 3 days followed by 100 mg once daily for 11 more days	CRP reduction, greater recovery in LVEF. No difference in length of hospital stay

Cavalli et al. (83) (2017)	Fulminant viral myocarditis (1), life-threatening myocarditis in AOSD (1) and T-cell lymphomas (1)	Case reports	Full recovery from cardiogenic shock, rapidly amelioration of cardiac function allowing weaning from mechanical circulatory and respiratory support
Cavalli et al. (84) (2016)			
Parisi et al. (85) (2017)			

ARAMIS (ongoing) https://ClinicalTrials.gov Identifier: NCT03018834	Acute myocarditis (120)	Double-blinded, randomized clinical trial Phase IIb of superiority ANAKINRA 100 mg/daily subcutaneously once a day vs placebo until hospital discharge, for a maximum of 14 days, in addition to standard care: ACE and Beta-blocker for 6 months	Results are awaited Primary outcome measures: no. of days alive free of any myocarditis complications defined as ventricular arrhythmias, HF, chest pain, ventricular dysfunction defined as LVEF < 50%, within 28 days post hospitalization

*n, number; AOSD, adult-onset Still disease; CAD, coronary artery disease; CFR, coronary flow reserve; CRP, C-reactive protein serym levels; FMD, flow-mediated reserve; HF, heart failure; HFpEF, heart failure with preserved ejection fraction; HFrEF, heart failure with reduced ejection fraction; LVEDVi, left ventricle end-diastolic volume index; LVEF, left ventricle ejection fraction; LVESVi, left ventricle end-systolic volume index; CMR, cardiac magnetic resonance; NSTEMI, non-ST-segment elevation myocardial infarction; ref, reference; STEMI, ST-segment elevation myocardial infarction; TTE, trans-thoracic echocardiography*.

*^a^VCU-ART and VCUART2 combined showed reduction in incidence of new HF with anakinra*.

*^b^An exploratory analysis showed a marked reduction in the incidence of lung cancer, as well as lung cancer mortality and total cancer mortality. The treatment was associated with a higher incidence of fatal infection than was placebo*.

*^c^Higher incidence of major adverse cardiac events at 12 months with anakinra*.

*^d^Chronic: non randomized study: anakinra (150 mg, *n* = 23) vs prednisolone (+5 mg over regular dose, *n* = 19) for 30 days*.

Besides dampening of inflammation, the beneficial effects of IL-1 blockade on cardiac function include amelioration of myocardial contractility. Early *ex vivo* studies with human atrial heart strips revealed that picomolar concentrations of IL-1 suppress contractile force (81). It was thus not unexpected that administration of anakinra to patients with refractory HF could improve exercise tolerance, while also dampening systemic pro-inflammatory mediators (51). IL-1 blockade with anakinra was also effective in the management of diastolic HF in a separate double-blind, placebo controlled trial (82). This direct beneficial effect of IL-1 inhibition on contractile function explains in part the near-instant clinical improvement observed in life-threatening cases of fulminant myocarditis, irrespective of the etiology (83–85). Similarly, in previous studies on patients with rheumatoid arthritis, anakinra promptly increased myocardial contractility within hours of a single dose administration (86, 87).

Given this dual efficacy on tissue inflammation and contractile dysfunction, anakinra seems a particularly attractive option for patients with inflammatory HF due to myocarditis. A double blind, randomized, phase-IIb placebo-controlled clinical trial of anakinra in patients with acute myocarditis is ongoing (ARAMIS-trial, https://www.clinicaltrials.gov/ct2/show/NCT03018834?term=anakinra&cond=myocarditis&rank=1). Experience is currently more limited with IL-1 blocking agents in the setting of chronic, VNM, and no clinical trials are presently evaluating this therapeutic option. However, VNM and DCM are histologically characterized by various degrees of inflammatory features, which precede and accompany fibrotic changes and drive both systolic impairment and arrhythmic complications. Prompt blockade of IL-1 can arrest the progression of uncontrolled inflammation, thus preventing fibrotic damage, curbing the arrhythmic burden, and restoring cardiac function. Also in light of a rapid onset of action and an excellent safety profile, IL-1 inhibition with anakinra represents a particularly suitable treatment option for conditions characterized by inflammatory HF, such as myocarditis with reduced LVEF.

## Conclusive Remarks

The inflammatory response in myocarditis spirals into a cycle of auto-inflammation, as intracellular contents released from dying myocardiocytes trigger the activation of the inflammasome and the uncontrolled release of IL-1 from neighboring cells. Selective and prompt pharmacologic inhibition of IL-1 dampens runaway inflammation and tissue damage, thus preventing arrhythmic complications and restoring cardiac function. Dual effectiveness against myocardial inflammation and contractile dysfunction renders IL-1 blockade a suitable therapeutic option for myocarditis.

## Author Contributions

GDL: conceived the hypothesis, contributed to the understanding of pathogenic mechanisms, clinical presentation and prognostic meaning of autoimmune myocarditis, and drafted the manuscript. GC: conceived the hypothesis, contributed to the understanding of biological effects of IL-1 and to the therapeutic usefulness of IL-1 in a broad spectrum of rheumatic diseases blockade, and critically revised the manuscript. CC: participated in previous studies focused on IL-1 blockade and myocarditis and critically revised the manuscript. MT: conceived the hypothesis and critically revised the manuscript. LD: conceived the hypothesis, critically revised the manuscript, and gave the approval of the final version.

## Conflict of Interest Statement

The authors declare that the research was conducted in the absence of any commercial or financial relationships that could be construed as a potential conflict of interest.
